# Significance of intranuclear angiotensin-II type 2 receptor in oral squamous cell carcinoma

**DOI:** 10.18632/oncotarget.26337

**Published:** 2018-11-27

**Authors:** Sayako Matsushima-Otsuka, Rina Fujiwara-Tani, Takamitsu Sasaki, Hitoshi Ohmori, Chie Nakashima, Shingo Kishi, Yukiko Nishiguchi, Kiyomu Fujii, Yi Luo, Hiroki Kuniyasu

**Affiliations:** ^1^ Department of Molecular Pathology, Nara Medical University, Kashihara, Nara 634-8521, Japan; ^2^ Jiangsu Province Key Laboratory of Neuroregeneration, Nantong University, Nantong, Jiangsu 226001, China

**Keywords:** AGTR2, renin-angiotensin system, ARB

## Abstract

The renin-angiotensin system (RAS) is implicated in the maintenance of blood pressure and in many other biological processes including tumorigenesis and metastasis formation. Angiotensin-II (A-II) type 2 receptor (AGTR2) seems to be involved in different types of cancer; its role, however, is still unclear. Here, we investigated the role of RAS, and specifically that of AGTR2, in oral squamous cell carcinoma (OSCC) progression. AGTR2 has opposite effect on vasodilation and blood pressure compared to AGTR1. In 23 OSCCs, we found that the *AGTR1/AGTR2* mRNA ratio was inversely associated with disease progression, while nuclear AGTR2 positivity was associated with disease progression. In the human OSCC cell lines HSC3 and HSC4, AGTR1 was associated with proliferation and invasion, while AGTR2 was associated with anti-apoptosis and anti-oxidative stress. Levels of nuclear AGTR2 confirmed by subcellular fractionation increased in hypoxic and hyperglycemic conditions, in which apoptosis and oxidative stress were suppressed and the redox status altered to reduction. Accumulation of nuclear AGTR2 by inhibition of extranuclear transportation decreased apoptosis and increased proliferation and invasion in HSC3 cells. Intratumoral angiotensin-II (but not serum angiotensin-II) levels were associated with stage and nuclear AGTR2 positivity. In OSCC cell lines, intracellular angiotensin-II was produced by themselves. Notably, losartan, an angiotensin receptor blocker, inhibited intracellular angiotensin-II production and AGTR2 nuclear localization to enhance the antitumoral effect of 5-FU in an OSCC tumor model. While the precise role of nuclear AGTR2 requires further examination, these data suggest that the intracellular angiotensin system might be a significant target for OSCC.

## INTRODUCTION

Angiotensin II (A-II) is converted into angiotensin by renin and causes vessel constriction and a rise in blood pressure by binding to the angiotensin type 1 receptor (AGTR1). Additionally, A-II induces cancer cell growth and invasion via AGTR1 [1]. In colorectal cancer (CRC) associated with diabetes, the activation of the renin-angiotensin system (RAS) is caused by hyperglycemic stimuli, which promote liver metastasis [2]. Thus, the activation of RAS is associated with malignant phenotypes through AGTR1 [1, 3]. On the other hand, A-II binds to angiotensin type 2 receptor (AGTR2) to cause effects opposite to those of AGTR1: vasodilatation and a fall in blood pressure [4]. Recent investigations have reported a role of AGTR2 in cardiovascular system, brain and renal function and also the modulation of various processes in organ development, cell differentiation, and tissue repair [5].

In addition to a function of RAS as a classic hormonal system, intracellular or intranuclear RAS has become the focus of recent attention [6]. In the nucleus, three RAS receptors, AGTR1, AGTR2, and MAS1, have been confirmed. These receptors, which are G protein coupled-protein receptors, are found in the nuclear membrane of human myocardial cells [7]. Subunits of AGTR2, including Gαq/11, Gαi/3, and Gβ, have been observed in the nuclei of canine atrial fibroblasts [8]. Furthermore, we have observed nuclear immunoreactivity to AGTR2 in CRCs [9]. It is thought that intracellular RAS interacts with extracellular (canonical) RAS [6, 10].

RAS, which acts systemically, plays a role in several types of cancer. AGT2R promotes tumor development, favoring both malignant cell proliferation and tumor angiogenesis [11]. However, AGTR2 also has an inhibitory effect on cell proliferation and invasion in CRC cells [9]. In the present study, we examined the effect of RAS in oral squamous cell carcinoma (OSCC) as a cancer in another organ site and of another histological type. OSCC has an incidence of 6,000 cases/year in Japan [12]. The main treatment for OSCC is surgical excision and radiation therapy and chemotherapy; however, the actual regimen is usually decided based also on functional, aesthetic, psychological, and social factors [13, 14]. Unfortunately, targeted therapies do not provide, yet, adequate improvement of OSCC survival [15]. Therefore, new molecular targets for effective treatment are needed. In this study, we attempted to clarify the role of RAS in OSCC and examined its possible use as a molecular target for OSCC treatment.

## RESULTS

### AGTR1 and AGTR2 mRNA expression in 23 OSCCs

**Figure 1:**
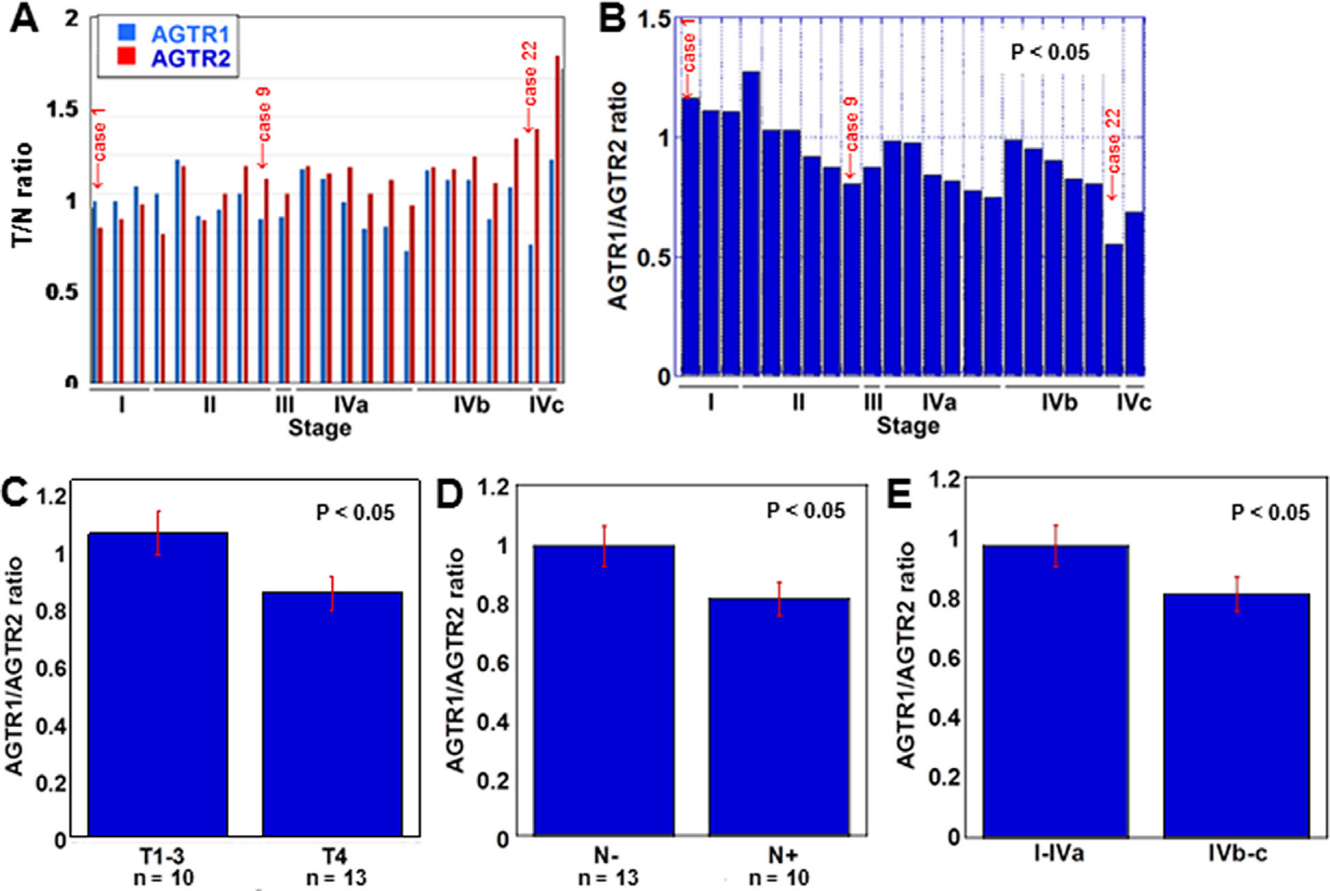
*AGTR1* and *AGTR2* mRNA expression in 23 OSCC cases (**A**) The expression of *AGTR1* and *AGTR2* was examined by qRT-PCR in tumor tissues and the coupled non-cancerous mucosae. The ratio of the expression in tumor to non-cancerous mucosa (T/N ratio) was sorted by the pathological stage of the samples [16]. (**B**) The ratio of *AGTR2* to *AGTR1* expression was sorted by the pathological stage of the samples. (**C–E**) *AGTR2*/*AGTR1* ratio according to tumor invasion (T factor, C), nodal metastasis (N factor, D) and pathological stage (E). Error bar, S.D.

We first examined the mRNA expression of *ATGR1* and *AGTR2* in 23 OSCC cases by qRT-PCR (Figure 1A and 1B). In Figure 1A, the mRNA expression of *ATGR1* and *AGTR2* is represented as tumor to non-tumor mucosal ratio (T/N ratio). The ratio of *AGTR2* increased as the stage progressed, while the ratio of *AGTR1* did not. Figure 1B indicates the ratio of *AGTR1* to *AGTR2* expression: such ratio was higher in early-stage cases than that in advanced-stage cases and was inversely associated with tumor expansion (Figure 1C), nodal metastasis (Figure 1D) and clinical stage (Figure 1E).

### Protein expression of AGTR2 and AGTR1 in 23 OSCCs

**Figure 2:**
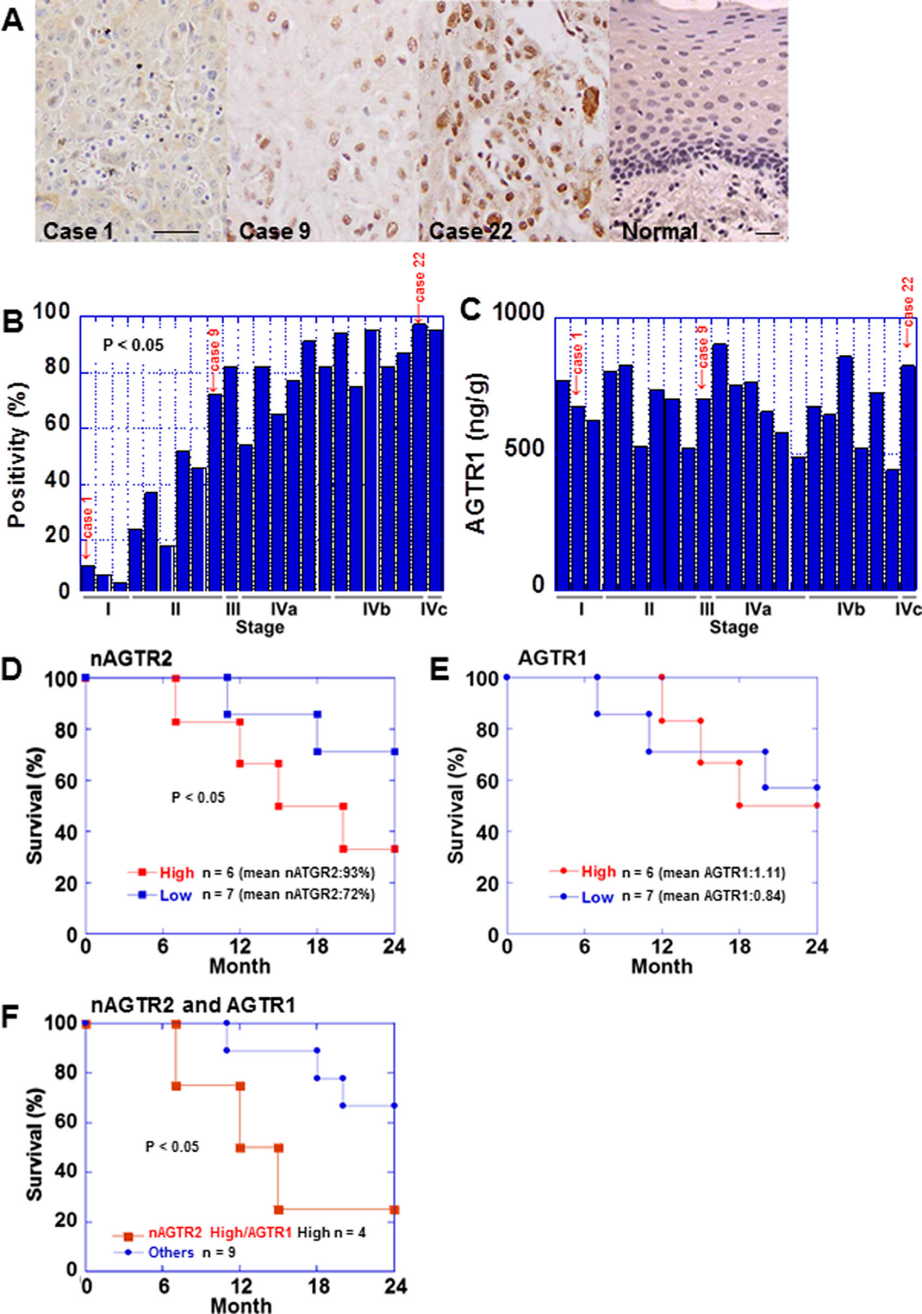
Expression of AGTR2 in 23 OSCC cases (**A**) AGTR2 immunohistochemistry showed the nuclear localization of AGTR2. Three representative images are shown. Case 1, 9% positivity, well differentiated SCC, stage I; case 9, 66% positivity, moderately differentiated SCC, stage II; case 22, moderately differentiated SCC, stage IVb. The same cases are highlighted in Figure 1A and 1B and in Figure 6A. Bar, 100 μm. (**B**) Percentage of cells with nuclear AGTR2 according to the pathological stage. (**C**) AGTR1 protein levels of the tumor tissues detected by ELISA according to the pathological stage. (**D, E**) Survival analyses of pStage IV OSCC cases. Cases were divided into 2 groups; 6 cases showing high value and 7 cases showing low value for nuclear ATGR2 positivity (D) or AGTR1 protein levels (E). (**F**) Survival analyses of the pStage IV cases divided into 2 groups; 4 cases showing high nuclear AGTR2 and high AGTR1 protein and 9 others. Statistical differences were calculated by Kaplan–Meier test.

Next, we examined AGTR2 protein expression in the same 23 OSCC cases by immunohistochemistry. AGTR2 immunoreactivity was detected in the nuclei of cancer and stromal cells (Figure 2A). AGTR2 immunoreactivity was not observed in non-cancerous epithelia and stromal cells. Then the nuclear AGTR2 is preferentially associated with tumors. The nuclear immunoreactivity of AGTR2 was more frequent and stronger in the advanced-stage cases than that in early-stage ones (Figure 2B and Table 1). Nuclear AGTR2 positivity was associated with tumor expansion, nodal metastasis and clinical stage (Table 1). AGTR1 protein levels in tumor tissues were also examined by ELISA in the 23 cases (Figure 2C). The AGTR1 protein levels were not associated with pT, pN or pStage.

**Table 1:** Relation of nuclear AGTR2 positivity with clinicopathological parameters

Parameter^1^		*n*	nATGR2 positivity (%)	*P*^2^
Age	–59	9	57 23	
	60–	14	58 27	NS
Sex	M	15	59 28	
	F	8	55 23	NS
pT	1–3	10	36 20	
	4	13	83 13	< 0.05
pN	0	13	47 18	
	1–3	10	83 14	< 0.05
pStage	I–III	10	36 20	
	IVa	6	75 13	
	IVb–IVc	7	89 8	< 0.05

^1^The tumors were classified according to the International Union Against Cancer TNM classification system [16].

^2^*P* value was calculated by non-parametric ANOVA test.

### Prognostic significance of nuclear AGTR2 expression

We next examined prognosis of pStage IV cases. The 13 cases of pStage IV were divided into 2 groups, 6 cases showing high value and 7 cases showing low value for nuclear ATGR2 positive or AGTR1 protein levels. Cases showing high values for nuclear AGTR2 positivity showed worse prognosis than that in cases showing low values (Figure 2D). In contrast, there was no prognostic difference between cases showing high expression for AGTR1 protein and those with low expression (Figure 2E). Moreover, the cases with high nuclear AGTR2 positivity and high AGTR1 protein showed worse prognosis than that in the other cases (Figure 2F).

Table 2A shows the results of univariate analysis of clinicopathological parameters. Nuclear AGTR2 positivity was the highest statistical significance followed by pathological stage (pStage). Table 2B shows results of multivariate analysis. Nuclear AGTR2 positivity was statistically significant, followed by pathological stage (pStage). *AGTR1* mRNA expression was not significant by univariate and multivariate analyses, whereas co-high values of nuclear AGTR2 positivity and *AGTR1* mRNA was significant by univariate analysis.

**Table 2:** Univariate and multivariate analyses of nuclear AGTR2 expression in 13 cases of Stage IV cases

A) Univariate analysis (log-rank trend test)		
	chi-square	*P* value
Stage (IVa, IVb-c)^1^	11.30	0.000120
pN (0, 1–3)^1^	14.00	0.002780
Nuclear AGTR2 (High, Low)^2^	18.70	0.001080
AGTR1 protein (High, Low)^3^ Nuclear AGTR2/AGTR1 protein (Both high, others)	13.30 14.56	0.278520 0.003420

B) Multivariate analysis (Cox’s proportional hazard model with stepwise method)				
	Hazard ratio	lower 95%	upper 95%	*P* value
Stage (IVa, IVb-c)^1^	3.587	2.658	12.065	0.01420
Nuclear AGTR2 (High, Low)^2^	2.145	1.025	4.228	0.03058
AGTR1 protein (High, Low)^3^ Nuclear AGTR2/AGTR1 protein (Both high, others)	0.031 1.564	0.002 0.823	0.784 2.447	0.46280 0.05224

^1^The tumors were classified according to the International Union Against Cancer TNM classification system [16].

^2^Nuclear AGTR2 positivity referred to Figure 2B and 2C.

^3^AGTR1 protein levels referred to Figure 2C and 2E.

### Expression of AGTR1 and AGTR2 in human OSCC cell lines

**Figure 3:**
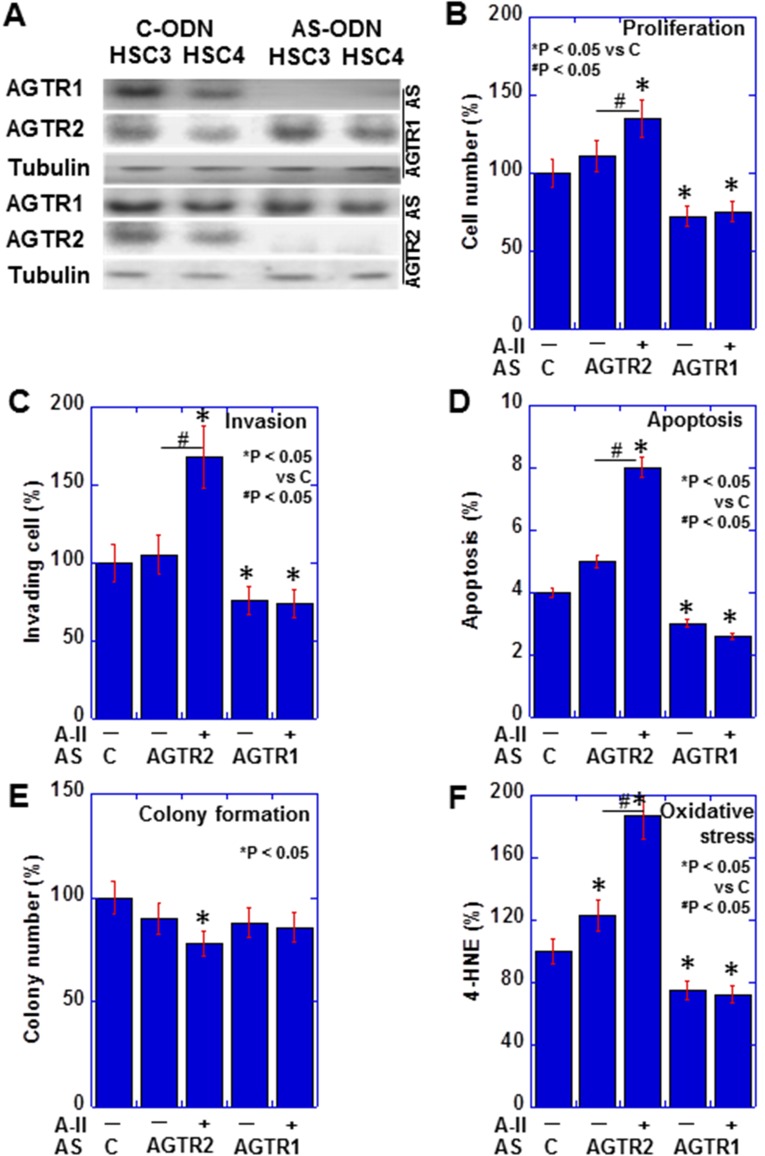
Effect of *AGTR1* and *AGTR2* knockdown in OSCC cells (**A**) The effect of antisense S-ODN on the expression of AGTR1 and AGTR2 was examined by immunoblotting in OSCC cells. (**B–F**) Effects of *AGTR1* or *AGTR2* knockdown on proliferation (B), invasion (C), apoptosis (D), colony formation (E), and oxidative stress (**F**) were examined with or without extrinsic A-II in HSC3 OSCC cells. Error bar, S.D.

We examined AGTR1 and AGTR2 protein expression in HSC3 (high metastatic) and HSC4 (low metastatic) human OSCC cell lines, by western blotting (Figure 3A). The levels of AGTR1 and AGTR2 were higher in HSC3 cells than in HSC4 cells in exposure to control ODN (C-ODN) with or without extrinsic A-II. Knockdown of *AGTR2* increased cell proliferation, invasion, apoptosis and oxidative stress, and suppressed colony formation with extrinsic A-II in HSC3 cells (Figure 3B–3F). In contrast, knockdown of *AGTR1* suppressed proliferation and invasion, and suppress apoptosis and oxidative stress with or without extrinsic A-II. HSC3 cells with AGTR2 knockdown showed significant alteration by extrinsic A-II in all parameters, whereas HSC3 cells with AGTR1 knockdown did not show any difference by extrinsic A-II.

### Nuclear AGTR2 in OSCC cells

**Figure 4:**
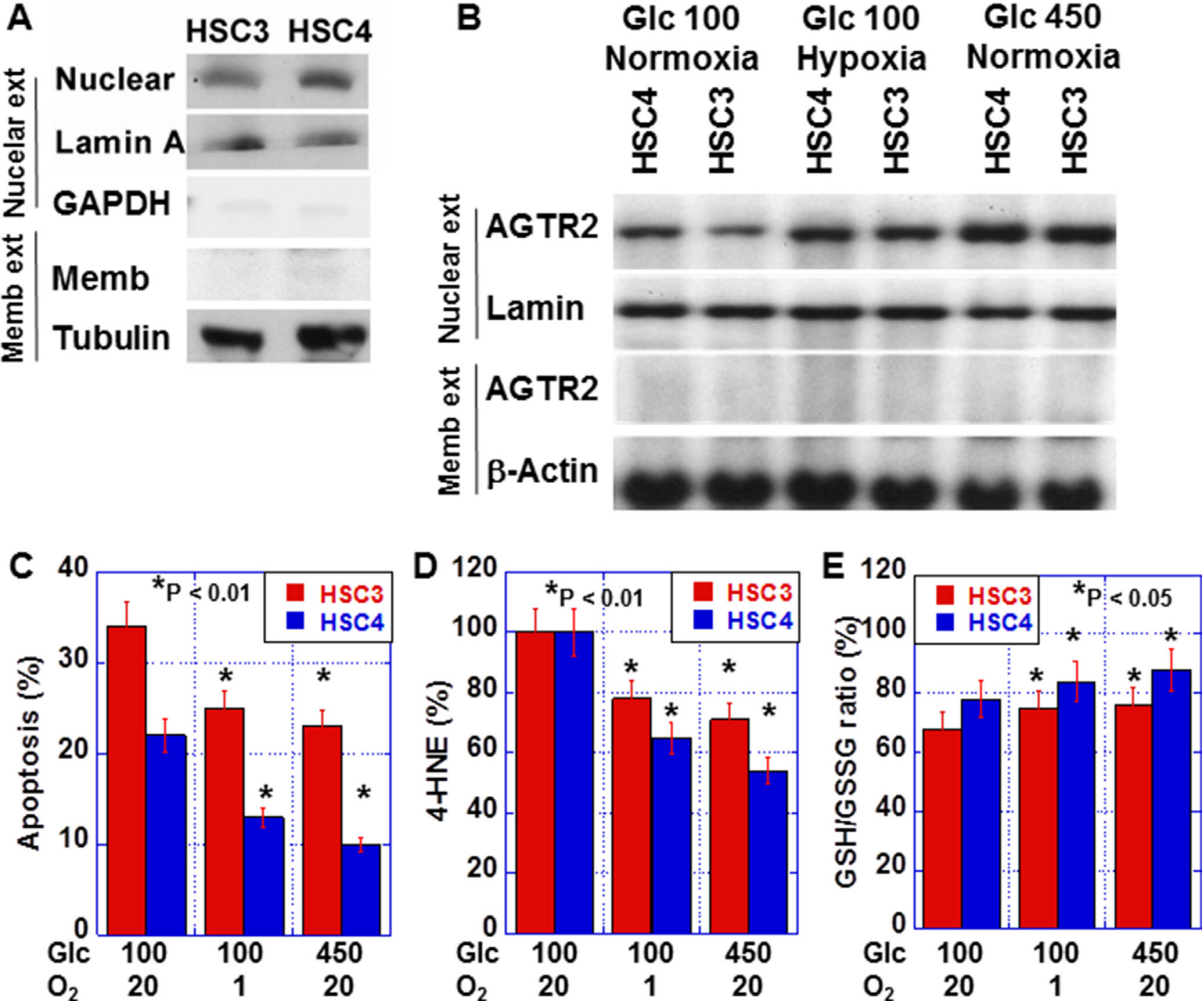
Effect of hypoxia and hyperglycemia on nuclear AGTR2 in OSCC cells (**A**) AGTR2 protein levels in nuclear and membrane fractions from OSCC cells. Glyceraldehyde-3-phosphate dehydrogenase (GAPDH) was examined as a cytoplastic marker. (**B**) Effect of hypoxia (1%) and hyperglycemia (450 mg/dl) on the nuclear localization of AGTR2 in OSCC cells. (**C–E**) Effect of hypoxia and hyperglycemia on 5-FU-induced apoptosis (C), oxidative stress (4-HNE; D) and redox status (GSH/GSSH ratio; E) in OSCC cells. Error bar, S.D. Nuclear ext, nuclear fracture; Memb ext, membrane fracture.

To confirm the nuclear localization of AGTR2, we conducted western blot assays after cell fractionation (Figure 4A). AGTR2 levels in membrane fractions were traceable in both HSC3 and HSC4 cells, whereas AGTR2 was detected clearly in the nuclear fraction. Nuclear AGTR2 was higher in HSC4 cells than that in HSC3 cells. As shown in Figure 4B, nuclear AGTR2 levels were increased in the two cell lines in hypoxic (O_2_, 1%) or hyperglycemic (glucose [Glc], 450 mg/dl) conditions in comparison with the cells in normoxic (O_2_, 5%) and normoglycemic (Glc, 100 mg/dl) conditions. In hypoxic or hyperglycemic condition, 5-FU-induced apoptosis in the two cell lines (Figure 4C). In these conditions, 4-HNE levels were decreased, while the GSH/GSSG ratios were increased (Figure 4D and 4E).

### Nuclear AGTR2 levels are associated with anti-apoptotic signals

**Figure 5:**
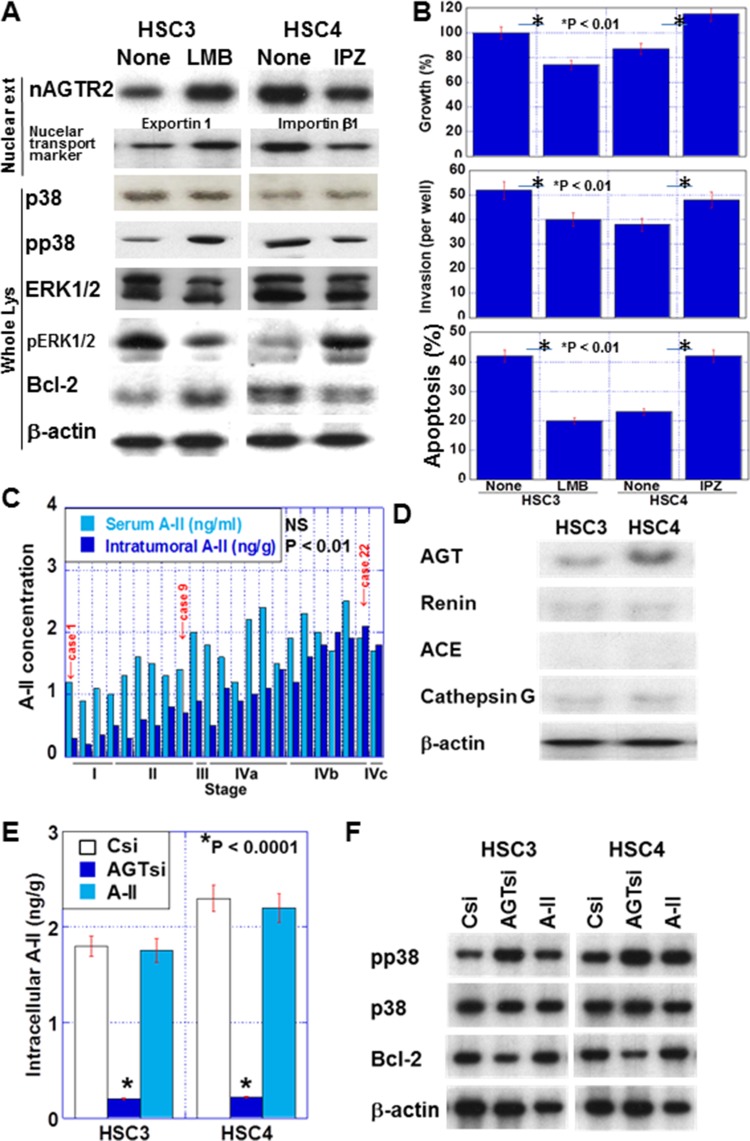
Effect of intracellular angiotensin II (A-II) in OSCC (**A**) Effect of IPZ and LMB on nuclear AGTR2 (nAGTR2), phosphorylation of p38 and ERK1/2, and Bcl-2 in OSCC cells. Exportin 1 and importin β1 were added as positive controls of LMB and IPZ treatment, respectively. (**B**) Effect of IPZ and LMB on proliferation, 5-FU-induced apoptosis, and invasion in OSCC cells. (**C**) The serum and intracellular A-II concentration in the 23 OSCC cases in relation to the pathological stage. (**D**) Expression of RAS-associated proteins in OSCC cells. The protein levels of angiotensinogen (AGT), renin, angiotensin I-converting enzyme (ACE) and cathepsin G were examined by immunoblotting. (**E** and **F**) Effect of *AGT* knockdown and extrinsic A-II on intracellular A-II concentration (E) and nuclear AGTR2 activation (F). In panel F, to assess nuclear AGTR2 activation, we examined the phosphorylation of p38 and Bcl-2 by immunoblotting in OSCC cells. Csi, control siRNA; AGTsi, AGT siRNA. Error bar, S.D. Nuclear ext, nuclear extract; Whole lys, whole cell lysate.

Nuclear AGTR2 levels were increased by LMB treatment in HSC3 cells and decreased by IPZ treatment in HSC4 cells (Figure 5A). Additionally, LMB-treated HSC3 cells showed decreased phosphorylation of ERK1/2, and increased phosphorylation of p38 and Bcl-2 levels, which suppressed cell growth, invasion, and apoptosis. In contrast, IPZ-treated HSC4 cells showed increased phosphorylation of ERK1/2, and decreased phosphorylation of p38 and Bcl-2, which enhanced cell growth, invasion, and apoptosis (Figure 5A and 5B).

### Intracellular A-II activates nuclear AGTR2 in OSCC

In the 23 OSCC cases examined above, intracellular A-II levels, but not serum A-II levels, were associated with the pathological stage (*P* < 0.01; Figure 5C) and nuclear AGTR2 levels (*P* < 0.001). In the two cell lines, AGT, renin and cathepsin G were expressed, possibly for intrinsic RAS activation (Figure 5D). In contrast, ACE was not detected in both cell lines. Knockdown of *AGT* reduced intracellular A-II, while extrinsic A-II did not affect intracellular A-II levels (Figure 5E). To examine the activation of AGTR2, the phosphorylation of p38 and Bcl-2 levels were detected in the two cell lines (Figure 5F). Knockdown of *AGT* increased p38 phosphorylation and decreased Bcl-2 levels. In contrast, extrinsic A-II did not affect p38 and Bcl-2 levels.

### Effect of losartan (LOS) on nuclear AGTR2 in OSCC cells

**Figure 6:**
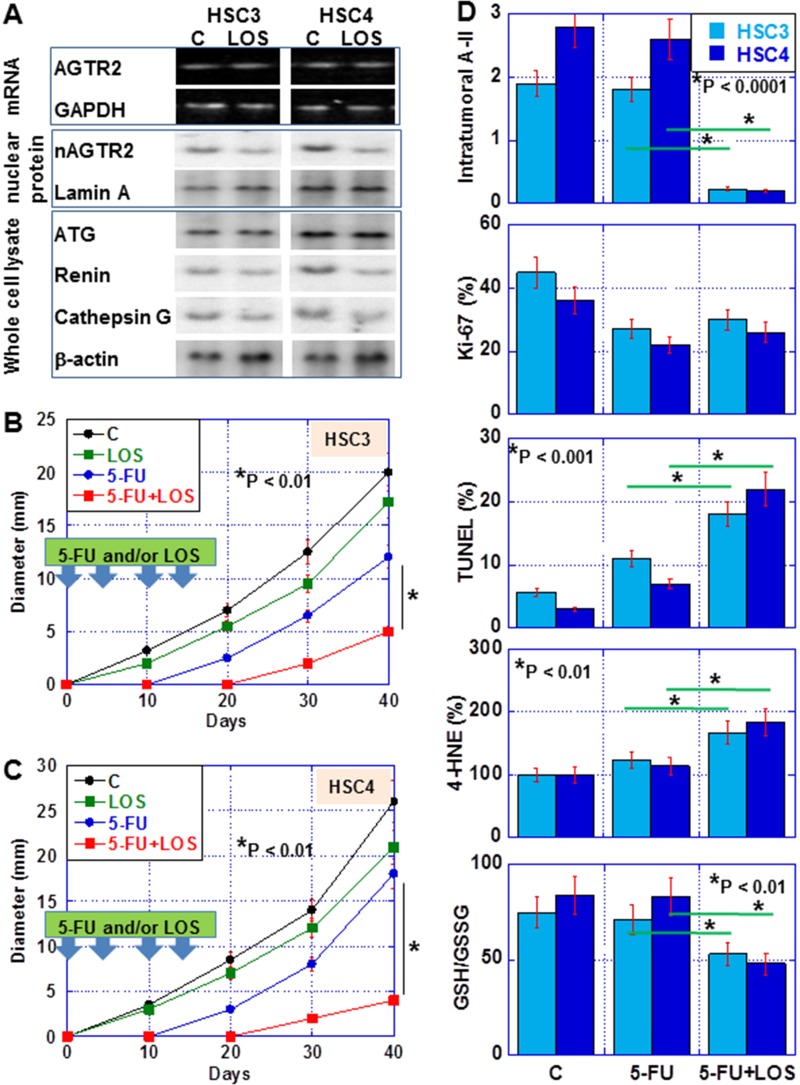
Effect of losartan (LOS) on tumor of OSCC cells in nude mice (**A**) Effect of LOS on intracellular RAS in OSCC tumors. HSC3 or HSC4 cells were inoculated into the back of nude mice (*n* = 6 in each group). Mice were treated with 5-FU (5 mg/kg body weight) and/or LOS (20 mg/Kg body weight) twice a week for two weeks. In LOS-treated tumors after 40 days, we measured *AGTR2* mRNA expression, and the protein levels of nuclear AGTR2 and RAS-associated proteins. (**B** and **C**) Effect of LOS on tumor growth of HSC3 and HSC4 cells, respectively, in nude mice. (**D**) Intratumoral A-II concentration, Proliferation (Ki-67), apoptosis (TUNEL), oxidative stress (4-HNE) and redox status (GSH/GSSG) in the tumors treated with 5-FU or 5-FU+LOS and untreated tumors. Error bar, S.D. Asterisk, statistical difference between 5-FU-treated mice and 5-FU+LOS-treated mice.

Finally, we examined the effect of losartan (LOS), an angiotensin receptor blocker (ARB), on nuclear AGTR2 in OSCC cells. In *in vitro* study, LOS decreased nuclear AGTR2 without affecting the mRNA levels of *AGTR2* in the two cell lines (Figure 6A). LOS also suppressed the levels of renin and cathepsin G but not those of AGT (Figure 6A).

We also examined the effect of LOS in the subcutaneous tumor model in nude mice (Figure 6B–6D). LOS decreased tumor growth by 14% and 17% in HSC3- and HSC4-derived tumors, respectively (Figure 6B and 6C). In HSC3-derived tumors, 5-FU alone inhibited tumor growth by 40%. In contrast, co-treatment with 5-FU and LOS inhibited tumor growth by 75%. In HSC4-derived tumors, 5-FU alone inhibited tumor growth by 21%. In contrast, co-treatment with 5-FU and LOS inhibited tumor growth by 87%.

In the tumors, LOS decreased intracellular A-II (Figure 6D). LOS did not enhances 5-FU-inhibited cell growth (Ki-67); however, apoptosis (TUNEL) was enhanced by LOS co-treatment with 5-FU with increase in oxidative stress (4-HNE) and oxidation (GSH/GSSG ratio) in the two cell lines.

## DISCUSSION

In this study, we found that the expression of *AGTR1* and *AGTR2* increased as disease progressed; however, *AGTR2* showed a more pronounced increment than *AGTR1* and, consequently, the *AGTR1* to *AGTR2* ratio decreased in advanced-stage cases. A previous study has shown that, upon stimulation of A-II, AT1R levels in the plasma membrane progressively decrease because of its internalization, whereas AGTR2 levels increase [17]. Contrarily, our immunohistochemistry data showed that AGTR2 levels increase in the nuclei and not on the cytoplasmic membrane. The nuclear localization but not membrane of AGTR2 was confirmed in OSCC cells by western blot analysis. AGTR2 plays an opposite role to AGTR1 in the cardiovascular system [4]. Notably, our data suggest that both AGTR1 and AGTR2 might have a pro-tumoral effect in OSCCs.

We have previously reported that hyperglycemia promotes A-II secretion in CRC cells, which enhances the progression of cancer through AGTR1 activation [2]. Here, we found that AGTR2 levels in the nucleus increase in hypoxic and hyperglycemic conditions and affect cell proliferation, invasion, and survival through the suppression of phosphorylated ERK1/2, and increase in phosphorylation of p38 and Bcl-2. Additionally, the increase in nuclear AGTR2 inhibits apoptosis through the decrease of oxidative stress levels and reduced state. Inhibition of mitogen-activated protein kinase (MAPK) by nuclear AGTR2 activation has been reported in human myocardial cells [18, 19]. In contrast, apoptosis is induced by a decrease in Bcl-2, increase in Bax and activation of caspase-3 upon activation of AGTR2 in the cytoplasmic membrane by extrinsic A-II [20]. Specifically, extracellular A-II activation stimulates the oxidative stress through the increase in p22phox and Nox-1, and the decrease in Nox-4 [21]. On the other hand, oxidative stress and nitric oxide increase mitochondrial AGTR2 levels, which suppress oxidative phosphorylation [22].

The difference of the roles of AGTR1 and AGTR2 is important issue in our study. We examined effect of knockdown of AGTR1 or AGTR2 with or without extrinsic A-II. AGTR1-associated (AGTR2 knockdown) phenotypes were significantly enhanced by extrinsic A-II, whereas AGTR2-associated (AGTR1 knockdown) phenotypes were not affected extrinsic A-II. A-II enhanced markedly AGTR1-associated phenotypes were pro-tumoral (proliferation and invasion) and anti-survival (apoptosis and oxidative stress). In contrast, AGTR2-associated phenotypes were anti-tumoral and pro-survival regardless of extrinsic A-II. These AGTR2-assciated phenotypes are resulted by nuclear AGTR2. To examine the phenotypes of nuclear AGTR2, we performed nuclear transportation inhibition assays. Nuclear accumulation of AGTR2 protein decrease 5-FU-induced apoptosis. Notably, serum A-II levels in OSCC did not associate with disease progression, while intra-tumoral A-II levels positively correlated with nuclear AGTR2 levels and were inversely correlated with the AGTR1-AGTR2 ratio. Our data might show that extrinsic A-II-dependent AGTR1 activation and intrinsic A-II activated nuclear AGTR2 play the complementary roles in OSCC progression. The coexpression of AGTR1 and nuclear AGTR2 provides advantage for OSCC, which is supported by the data of the survival analyses. It is necessary to investigate by increasing cases to confirm the clinical significance of the findings.

AGTR2 level was not affected by extracellular A-II but it was inhibited by knockdown of *AGT*. Therefore, nuclear AGTR2 is not activated by extracellular A-II but by intracellular A-II, produced by AGT, renin, and cathepsin G in cancer cells. On this note, it has been reported that AGTR2 nuclear transport, promoted by hyperglycemia, also increases intracellular A-II [7]. Furthermore, hyperglycemia promotes proliferation and invasion in cancer cells upon activation of AGTR1 by extracellular A-II, and suppresses apoptosis, decreases the levels of reactive oxygen species, and maintains the reduced redox state through the increase of intracellular A-II and activation of nuclear AGTR2 [2, 9]. Consequently, it is thought that AGTR2 has a cell-protective role in OSCC cells and reduces cell damages caused by extracellular A-II [10, 23].

Nuclear localization of AGTR2 was confirmed in our data; however, the mechanism of nuclear transportation was not unclear. Nuclear transportation of AGTR2 or A-II is reported [2, 7, 9]; however, AGTR2 and A-II proteins do not possess common nuclear transportation domains by search in NCBI data base (data not shown). Our data suggest that AGTR2 might be transported by the active transport system with importin/exportin. In contrast, since A-II transportation was seemed not to be affected by treatment with LMB or IPZ, nuclear transportation of A-II might be independent of the importin/exportin system. Further examination should be needed in this issue.

We examined the effects of losartan (LOS), an ARB used as a RAS antagonist, on nuclear AGTR2. LOS did not affect AGTR2 mRNA expression but suppressed its nuclear transport. LOS also reduced the intracellular A-II levels by repressing AGT, renin, and cathepsin G and importantly, cell survival was limited by the increased oxidative stress and enhanced oxidized redox state. Inhibition of extracellular RAS through AGTR1 blockage suppressed nuclear AGTR2. In the mouse tumor model, treatment with LOS alone was associated with weak suppression of tumor growth. However, cotreatment with LOS and the chemotherapeutic drug 5-FU provided synergistic growth inhibition. Therefore, ARBs, by inhibiting both extracellular and intracellular RAS, might increase the effectiveness of chemotherapy in OSCC.

In conclusion, in the present study, we demonstrated that activation of intracellular RAS by nuclear AGTR2 might promote OSCC progression and RAS might be a novel therapeutic target.

## MATERIALS AND METHODS

### Surgical specimens

Pathological diagnosis and clinical data were reviewed in 23 patients with OSCCs that had been diagnosed in the Department of Molecular Pathology, Nara Medical University (Japan) from 2008 to 2016. The primary tumor site was tongue (12 cases), gingiva (6 cases), buccal mucosa (4 cases) and other (1 case). The tumors were classified according to the International Union Against Cancer TNM classification system [16]. Because written informed consent was not obtained, identifying information for all samples was removed before analysis for strict privacy protection (unlinkable anonymization). All procedures were performed in accordance with the Ethical Guidelines for Human Genome/Gene Research enacted by the Japanese Government, which was approved by the Ethics Committee of Nara Medical University (Approval Number 937).

### Animal model

BALB/c nu/nu athymic mice (male, 4 weeks old) purchased from Japan SLC Inc. (Shizuoka, Japan) were used to generate a subcutaneous tumor model. The mice were maintained according to the institutional guidelines approved by the Committee for Animal Experimentation of Nara Medical University, in accordance with the current regulations and standards of the Ministry of Health, Labor, and Welfare (Approval number 11569).

A single-cell suspension (1 × 10^7^ cells) in Hanks’ balanced saline solution (Wako Pure Chemical Industries, Ltd., Osaka, Japan) was injected into the subcutaneous layer in the back of mice. The size of tumors was monitored every 10 days. The mice were euthanized 40 days after inoculation and isolated tumor tissues were subjected to protein analysis. 5-fluorourasil (5-FU; 5 mg/kg body weight, Wako) and losartan (LOS; 20 mg/Kg body weight, Wako) were administrated intraperitoneally twice a week for two weeks after tumor cell inoculation.

### Cell culture and reagents

HSC3 and HSC4 human OSCC cell lines were purchased from Dainihon Pharmaceutical Co. (Tokyo, Japan). Cells were maintained in Dulbecco’s modified essential medium (DMEM; Wako) containing 10% fetal bovine serum (Sigma Chemical Co., St. Louis, MO, USA) under 5% CO_2_ and 37°C conditions. The glucose concentration in the regular medium was 100 mg/dl. To mimic hyperglycemic conditions, we used DMEM with high glucose concentration (450 mg/ml, WAKO). A-II (1 ng/ml, Abgent, San Diego, CA, USA), LOS (2 μg/ml, Wako), importazole (IPZ, 20 μM, Sigma) and leptomycin B (LMB, 2 μM, Sigma) were used for cell treatment.

### Hypoxic culture

Hypoxic conditions were generated by using the Bionix Hypoxic Culture kit (Sugiyama-gen Co. Ltd., Tokyo, Japan). In accordance with the manufacturer’s instructions, the O_2_ concentration in the culture pouch was kept at 1%.

### Cell growth, apoptosis, and *in vitro* invasion assay

Cells were seeded at a density of 10,000 cells per well in 12-well tissue culture plates. Cell growth was assessed by cell counting using an cytometer (Sysmecs, Kobe, Japan). Apoptosis was induced by 5-FU (5 μg/ml) and assessed by staining with the Hoechst 33258 fluorescent dye (Wako). The number of apoptotic cells was determined by examining 1,000 cells [24]. A modified Boyden chamber assay was performed to examine the *in vitro* invasion of colon cancer cells [24]. The experiments were performed three times.

### Antisense phosphorothioate(S)-oligodeoxynucleotide assay

The 16-mer antisense S-oligodeoxynucleotides (ODNs) for the first sixteen nucleotides of AGTR1 and AGTR2 cDNAs were synthesized (Sigma-Genosys, Ishikawa, Japan). The sequences were AGTR1: 5′-AAG AGT TGA GAA TCA T-3′ (GenBank: BC022447.1) and AGTR2: 5′-TGG AGT TGC CCT TCA T-3′ (GenBank: NM_000686.4). A random 16-mer sequence was used as negative control. For treatment of the cells, 6 μM of S-ODNs were used, for 48 h.

### Colony formation assay

Cells (5 × 10^4^) were seeded onto a 3.5 cm-dish and cultured with regular medium for three days and exposed to S-ODNs. The formation of cell colonies was observed by phase contrast microscopy. The experiments were performed three times.

### Short interfering RNA (siRNA)

Stealth Select RNAi for angiotensinogen (AGT; AGTsi) and control siRNA (Csi) were purchased from Invitrogen (Carlsbad, CA, USA). siRNAs (20 nM) were transfected in the cells using Lipofectamine 2000 (Invitrogen) according to the manufacture’s recommendations. RNA interference was confirmed by quantitative reverse transcription-polymerase chain reaction (qRT-PCR).

### qRT-PCR

The extraction of total RNA was carried out using a RNeasy Mini Kit (Qiagen Genomics, Bothell, WA, USA) and cDNA (1 μg) was synthesized with the ReverTra Ace-α-RT Kit (Toyobo, Osaka, Japan). qRT-PCR was performed using the StepOne Real-Time PCR System (Applied Biosystems, Foster City, CA, USA) using the Fast SYBR Green Master Mix (Applied Biosystems) and analyzed using the relative standard curve quantification method [25]. PCR conditions were set according to the manufacturer’s instructions. Actin B (*ACTB*; GenBank accession No. NM 001101) was used as internal control. Each amplification reaction was evaluated by a melting curve analysis. For the visualization the PCR products, agarose gel electrophoresis and ethidium bromide staining were performed. Primer sets were as follows: AGTR1(GenBank accession No. BC022447.1), forward 5′-GCA CAA TGC TTG TAG CCA AA-3′ and reverse 5′-GGG TTG AAT TTT GGG ACT CA-3′; and AGTR2 (GenBank accession No. NM_000686.4), forward 5′-TTC CCT TCC ATG TTC TGA CC-3′ and reverse 5′-AAA CAC ACT GCG GAG CTT CT-3′.

### Protein extraction

Fresh tissues (approximately 10 mm^3^) were obtained, frozen in liquid nitrogen and stored at –80°C. Frozen tumor tissues were sonicated in lysis buffer (50 mM Tris HCl, pH 7.5, 5 mM EDTA, 1 mM EGTA, 2% Nonidet P-40, 10 µg/ml leupeptin, 50 µg/ml phenylmethylsulfonyl fluoride), and centrifuged (5000 × *g*). The supernatant was used for further examinations. Cell fractions were extracted by processing the cells with a Cell Fractionation Kit (Abcam, Cambridge, MA, USA), according to the manufacture’s instruction. The blood was rapidly centrifuged at 500 × *g*, for 4 min and at 4°C and the supernatant obtained was used as serum.

### Immunoblot analysis

Fifty micrograms of lysates were subjected to immunoblot analysis using 12.5% SDS-polyacrylamide gels followed by electrotransfer onto nitrocellulose membranes [26]. The membranes were incubated with a primary antibody and then with a peroxidase-conjugated anti-goat IgG antibody (Medical and Biological Laboratories, Nagoya, Japan). The following antibodies were used: A-II (Serotec Ltd., Oxford, UK), AGT (R&D Systems Inc., Minneapolis, MN, USA), AGTR1 (Assay Designs, Inc, Ann Arbor, MI, USA), AGTR2 (Assay Designs), renin (AnaSpec, Inc., San Jose, CA, USA), angiotensin I-converting enzyme (ACE; Life Span Biosciences, Inc., Salt Lake City, UT, USA), cathepsin G (Santa-Cruz Biotechnology, Inc., Santa Cruz, CA, USA), extracellular signal–regulated kinase (ERK)1/2, phosphorylated ERK1/2 (pERK1/2, Santa Cruz), p38 (Santa Cruz), phosphorylated p38 (pp38, Santa Cruz), importin β1/KPNB1, expotin 1/CRM1, glyceraldehyde-3-phosphate dehydrogenase (GAPDH) (Abcam), and B-cell lymphoma 2 (Bcl-2; DAKO Corp, Carpinteria, CA, USA). Additionally, we used antibodies for tubulin, β-actin and lamin (Zymed Laboratories Inc., South San Francisco, CA, USA) to detect the corresponding proteins for normalization. The immune complexes were visualized using an Enhanced Chemiluminescence Western-blot detection system (Amersham, Aylesbury, UK).

### Enzyme-linked immunosorbent assay (ELISA)

The concentration of A-II (Phoenix Pharmaceuticals, Inc., Belmont, CA), 4-hydroxynonenal (4-HNE; R&D Systems Inc., Minneapolis, MN, USA), glutathione/glutathione disulfide (GSH/GSSG; Dojindo, Kumamoto, Japan) and AGTR1 (Life Span Biosciences) were measured in cell lysates or nuclear extracts using ELISA kits according to the manufacturer’s instructions [27, 28].

### Immunohistochemistry

Consecutive 4-μm sections were immunohistochemically stained using the immunoperoxidase staining technique described previously [29]. For antigen retrieval, an autoclave treatment (121°C, 40 min) was done. Anti-AGTR2 (Assay Designs) or -Ki-67 (DAKO Corp.) antibodies (0.5 µg/ml) were used as primary antibodies. Secondary antibodies (Medical & Biological Laboratories, Nagoya, Japan) were used at a concentration of 0.2 µg/ml. Specimens were color-developed with diamine benzidine hydrochloride (DAB; DAKO Corp.). Meyer’s hematoxylin (Sigma Chemical Co.) was used for counterstaining. Immunostaining of all specimens was performed simultaneously to ensure same antibody reaction and DAB exposure conditions.

### Terminal deoxynucleotidyl transferase dUTP nick end labeling (TUNEL) assay

Apoptotic cells were detected by TUNEL assay using the *In Situ* Cell Death Detection Kit, POD (Roche Diagnostics, Indianapolis, IN, USA). The percent frequency of TUNEL-positive cells was calculated from the ratio of positive nuclei to 500 examined nuclei.

### Statistical analysis

Statistical analyses of experimental data were performed using the Mann-Whitney *U* test, Kruskal-Wallis test with Dunn’s multiple comparison test (non-parametric ANOVA), unpaired *t* test with Welch correction, and chi-squared test. Non-parametric correlation was examined by Spearman rank correlation test. Survival analysis was performed using the Kaplan–Meier method along with the log-rank test. Univariate and multivariate analyses were performed using the log-rank trend test and the Cox’s hazard model, respectively (SPSS Statistics, IBM Japan, Tokyo, Japan). Statistical significance was defined as a two-sided *P* value lower than 0.05.

## Abbreviations

RAS: renin-angiotensin system
A-II: angiotensin-II
AGTR1: angiotensin-II type 1 receptor
AGTR2: angiotensin-II type 2 receptor
OSCC: oral squamous cell carcinoma
CRC: colorectal cancer
5-FU: 5-fluorourasil
LOS: losartan
IPZ: importazole
LMB: leptomycin B
ODN: oligodeoxynucleotide
AGT: angiotensinogen
ACE: angiotensin I-converting enzyme
ERK: extracellular signal–regulated kinase
BCL-2: B-cell lymphoma 2
4-HNE: 4-hydroxynonenal
GSH: glutathione
GSSG: glutathione disulfide
TUNEL: terminal deoxynucleotidyl transferase dUTP nick end labeling
